# Joint association of daily sitting time and sleep duration with constipation among the US population

**DOI:** 10.3389/fnut.2025.1548455

**Published:** 2025-03-13

**Authors:** Yu-Jun Xiong, Hua-Zhao Xu, Xiang-Da Meng, Xing-Yun Zhu, Tian Lv, Jian-Fei Huang

**Affiliations:** ^1^Department of Gastroenterology, Beijing Hospital, National Center of Gerontology, Institute of Geriatric Medicine, Chinese Academy of Medical Sciences, Beijing, China; ^2^Hospital Administration Office, Beijing Hospital, National Center of Gerontology, Institute of Geriatric Medicine, Chinese Academy of Medical Sciences, Beijing, China; ^3^Department of Hernia and Abdominal Wall Surgery, Peking University Peoples’ Hospital, Beijing, China; ^4^Department of Endocrinology, Beijing Jishuitan Hospital, Beijing, China; ^5^Department of Neurology, Zhuji Affiliated Hospital of Wenzhou Medical University, Zhuji, China; ^6^Department of Proctology Surgery, Shaoxing People’s Hospital, Shaoxing, China

**Keywords:** constipation, daily sitting time, sleep duration, association, NHANES

## Abstract

**Background:**

This study aimed to investigate the independent and combined effects of prolonged daily sitting time and sleep duration on the risk of constipation, using data from the National Health and Nutrition Examination Survey (NHANES) 2005–2010.

**Methods:**

A total of 6,894 participants were included in the analysis. Daily sitting time and sleep duration were self-reported and categorized as short (<7 h/day) or long (≥7 h/day). Constipation was defined based on stool consistency and frequency using the Bristol Stool Form Scale. Multivariable logistic regression models were used to estimate the odds ratios (ORs) for constipation. A restricted cubic spline analysis was applied to assess the dose–response relationships.

**Results:**

Participants with prolonged daily sitting time and short sleep duration showed a higher risk of constipation compared to those with long sleep duration and short sitting time. In multivariable-adjusted models, long sitting time was positively associated with an increased risk of constipation (OR = 1.424; 95% CI, 1.114–1.821), while long sleep duration was associated with a lower constipation risk (OR = 0.725; 95% CI, 0.553–0.952). The joint analysis revealed that the combination of short sleep duration and long sitting time was associated with the highest constipation prevalence (OR = 1.975; 95% CI, 1.378–2.833).

**Conclusion:**

Both prolonged sitting time and insufficient sleep were associated with an increased risk of constipation, especially when combined. These findings underscore the importance of adopting healthier sleep habits and reducing sedentary behavior to lower constipation risk.

## 1 Introduction

Constipation is a prevalent gastrointestinal disorder that affects a significant portion of the population, with various factors influencing its onset, including diet, hydration, physical activity, and sleep (1). Among these, sleep duration has been the subject of growing research interest (2–4). Sleep deprivation is known to alter gut microbiota dysbiosis and gastrointestinal motility, which may influence bowel habits and stool consistency (5).

Sedentary behavior has been implicated in numerous adverse health outcomes, including metabolic disorders, cardiovascular disease, and certain cancers (6, 7). Extended sitting time is usually associated with reduced physical activity, which is a known risk factor for constipation (8). Physical activity promotes gastrointestinal motility, while sedentary behavior may slow down transit time, increasing the likelihood of hard stools and infrequent bowel movements. Despite these plausible connections, the direct relationship between long daily sitting time and constipation has received little attention in the scientific literature. This lack of research creates a critical gap, especially considering the modern lifestyle where prolonged sitting—whether at work, during commutes, or through recreational screen time—has become increasingly common.

However, previous research has primarily focused on the independent effects of sleep duration and daily sitting time on constipation, with limited attention to their combined impact. The interaction between prolonged sitting and sleep duration is particularly important, as these factors may jointly exacerbate the risk of constipation. Since both sitting time and sleep are key components of daily living, understanding how they work together to influence bowel health could provide valuable insights for developing preventive strategies. This study aims to address this research gap by exploring the independent and combined associations of long daily sitting time and sleep duration with the risk of constipation. Filling this gap is crucial for a more comprehensive understanding of how lifestyle factors contribute to gastrointestinal health.

## 2 Materials and methods

### 2.1 Study design and participants

The NHANES initiative employs a refined and intricate methodology to periodically select a representative sample of the U.S. population. Its primary objective involves evaluating the health and nutritional status of individuals in the United States (9). To uphold ethical standards, the survey has garnered approval from The National Center for Health Statistics Institutional Review Board. Furthermore, prior to their inclusion in the study, all participants have willingly provided written informed consent. NHANES encompasses a broad spectrum of data, including demographics, dietary patterns, medical examination results, laboratory findings, and responses to questionnaires (10).

**Figure 1:**
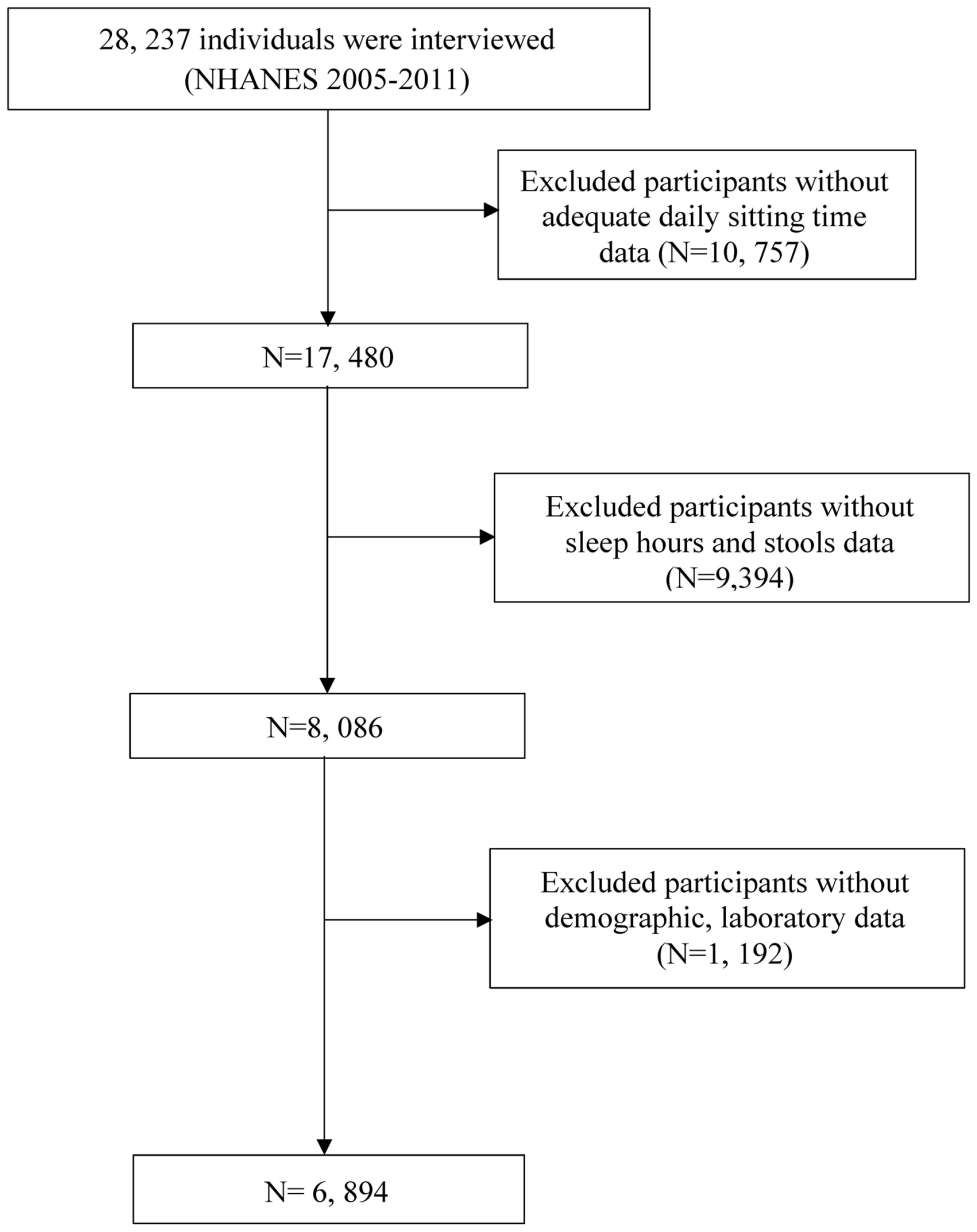
Flowchart of participant screening.

Throughout the NHANES 2005–2010 cycle, the study encompassed 28,237 participants, following the application of exclusion criteria. Exclusion criteria involved missing hypertension, missed BMI data, miss education data, missed alcohol status, missed WBC data, missed smoke status, missed DM data, missed poverty data, missed uric acid data, missed HbA1c data, missed bilirubin data, missed CVD data, and other missing covariate data (Figure 1).

### 2.2 Definition of constipation

In the NHANES dataset, constipation was determined using either stool frequency or stool consistency, as captured in the Bowel Health Questionnaire (2005–2010) (11). Participants assessed their typical fecal consistency using the Bristol Stool Form Scale (BSFS), which categorizes stool into seven types. Constipation was defined as BSFS Type 1 (hard, separate lumps resembling nuts) or Type 2 (lumpy, sausage-like stools), while non-constipation was classified as Type 3 (sausage with cracks on the surface), Type 4 (smooth and soft, resembling a sausage or snake), Type 5 (soft blobs with clear-cut edges), Type 6 (fluffy pieces with ragged edges) or 7 (entirely liquid with no solid pieces) (12, 13).

### 2.3 Definition of daily sitting time and sleep hours

In our study, daily sitting time served as the exposure variable, assessed through the question: “How much time do you usually spend sitting or reclining on a typical day?” This referred to the total waking hours spent sitting or reclining at work, home, or school, including activities such as sitting at a desk, socializing, commuting by car, bus, or train, reading, playing cards, watching TV, or using a computer (14). Based on previous research, sitting time was categorized into two groups: short (<7 h) and long (≥7 h) (15).

Sleep duration for NHANES participants was assessed via a questionnaire administered by trained interviewers using a computer-assisted personal interview system. Participants were asked, “How much sleep do you usually get at night on weekdays or workdays?” (16, 17) Based on the responses, sleep duration was self-reported as the number of hours slept within a 24-h period on weekdays and categorized into two groups: short sleep duration (<7 h) and long sleep duration (≥7 h) (18).

### 2.4 Covariate

According to prior research and clinical experts, potentially confounding and modifying variables were identified as follow: age group (<60, ≥60 years), sex (male or female), race (White, Black, other), education (less than high school, high school, more than high school), poverty-to-income ratio (PIR), BMI (calculated based on the ratio of weight in kilograms to height in meters squared), smoking status (current, previous, or never). Clinical indicators such as uric acid, WBC, HbA1c were measured in the NHANES laboratory. The diagnosis of cardiovascular disease (CVD) was established by self-reported physician diagnoses obtained during an individual interview using a standardized medical condition questionnaire. Hypertension was defined as self-reported hypertension, a systolic blood pressure (SBP) of ≥140 mmHg, a diastolic blood pressure (DBP) of ≥90 mmHg, or the use of antihypertensive medications (19). Alcohol drinking status was classified into four distinct categories, reflecting their alcohol consumption patterns: Never drinkers (lifetime abstainers), former drinkers (abstinent within the past year), moderate drinkers (1 or 2 drinks per day for females/males, respectively), and heavy drinkers (>1 or > 2 drinks per day for females/males, respectively, and/or frequent binge drinking) (20, 21).

### 2.5 Statistical analysis

The baseline characteristics of participants were summarized and compared between *H. pylori*-infected and uninfected patients. Continuous variables were expressed as mean (±SD) and compared using either a t-test or Wilcoxon rank-sum test, based on the outcome of the Kolmogorov–Smirnov normality test. Categorical variables were presented as frequency (percentage) and compared using the Chi-square test.

Additionally, the potential nonlinear connections between sleep hours, daily sitting time and constipation were explored using weighted restricted cubic spline (RCS) curves. These curves were positioned at specific percentiles (10, 50, and 90%) within the sleep hours and daily sitting time distribution (22). Weighted multivariable-adjusted logistic regression analyses were applied to determine the odds ratio (OR) alongside a 95% confidence interval (CI) for assessing the relationship between daily sitting time, sleep hours and constipation. To avoid overadjustment and optimize data utilization for variables, two models were developed: model 1 adjusted for age, sex, and races, and model 2 encompassed BMI, education, smoke, alcohol drink, diabetes mellitus, hypertension, poverty, uric acid, CVD, WBC and HbA1c in addition to the adjustments in model 1.

To assess the combined effects of sleep duration and daily sitting time on the likelihood of constipation, we generated dummy variables representing four categories based on the cross-tabulation of sleep duration (short and long) and sitting time (short and long). Using short sitting time (<7 h) and long sleep duration (≥7 h) as the reference group, we examined the joint associations between these factors and the risk of constipation.

To determine whether the associations between these factors and the risk of constipation varied by demographic characteristics, we assessed potential effect modification by age (<60 vs. ≥60 years), sex (women vs. men), and BMI (≥30 vs. <30), after adjustment for covariates like races, education, smoke, alcohol drink, diabetes mellitus, hypertension, poverty, uric acid, CVD, WBC and HbA1c.

The additive interaction between sleep duration (<7 h, ≥7 h) and daily sitting time (<7 h, ≥7 h) in association with constipation was measured by whether the estimated joint effect of two factors was greater than the sum of the independent effect of sleep duration and daily sitting time. Relative excess risk due to interaction (RERI), attributable proportion of interaction (AP) and synergy index (S) were used to assess the additive interaction. When the confidence interval of RERI and AP contained 0 and the confidence interval of S contained 1, there was no additive interaction.

A two-sided *p* < 0.05 was considered statistically significant. All analyses were performed using SPSS version 26.0 (IBM Corp, Armonk, NY) and R (version 4.3.2) (23, 24).

## 3 Results

### 3.1 Study participants and baseline characteristics

In the final cohort, 6,894 American adults were included, among whom 494 (7.2%) participants were classified as constipated (Figure 1; Table 1). The mean age of the constipation group was 45.859 ± 0.772 years, significantly lower than that of the non-constipation group (47.709 ± 0.405 years, *p* = 0.014). The proportion of male participants was significantly lower in the constipation group (32.2%) compared to the non-constipation group (51.3%, *p* < 0.001). Individuals with constipation exhibited a significantly lower mean BMI (27.731 ± 0.412 vs. 28.993 ± 0.118, *p* = 0.009) and a lower poverty-income ratio (2.922 ± 0.106 vs. 3.211 ± 0.053, *p* = 0.001). Educational attainment also differed significantly between groups (*p* = 0.010), with a higher percentage of individuals in the constipation group having less than a high school education (17.4% vs. 15.7%).

**Table 1:** Weighted characteristics between constipated and non-constipated participants.

	Overall (*n* = 6,894)	Non-constipation (*n* = 6,400)	Constipation (*n* = 494)	*p* value
Age (years)	47.578 (0.401)	47.709 (0.405)	45.859 (0.772)	0.014
Sex (Male %)	3,519 (50.0)	3,352 (51.3)	167 (32.2)	< 0.001
BMI (kg/m^2^)	28.904 (0.107)	28.993 (0.118)	27.731 (0.412)	0.009
Race (%)				0.183
Black	1,216 (9.479)	1,117 (9.284)	99 (12.023)	
Other	1848 (15.520)	1715 (15.504)	133 (15.727)	
White	3,830 (75.002)	3,568 (75.212)	262 (72.250)	
WBC (1,000 cells/ul)	7.197 (0.038)	7.190 (0.037)	7.292 (0.135)	0.434
Poverty income ratio	3.191 (0.055)	3.211 (0.053)	2.922 (0.106)	0.001
Education (%)				0.010
Less than high school	1,642 (15.830)	1,511 (15.711)	131 (17.382)	
More than high school	3,638 (61.334)	3,410 (61.844)	228 (54.668)	
High school	1,614 (22.835)	1,479 (22.445)	135 (27.950)	
Albumin (g/L)	42.756 (0.072)	42.786 (0.074)	42.371 (0.149)	0.013
HbA1c (%)	5.594 (0.017)	5.595 (0.017)	5.585 (0.053)	0.845
Total bilirubin (mg/dL)	0.783 (0.006)	0.788 (0.006)	0.718 (0.014)	< 0.001
Uric acid (mg/dL)	5.528 (0.029)	5.560 (0.029)	5.100 (0.078)	< 0.001
BUN (mg/dL)	13.277 (0.127)	13.302 (0.128)	12.956 (0.216)	0.084
CVD = yes (%)	850 (9.1)	791 (9.1)	59 (8.3)	0.416
Smoke (%)				0.008
Former	1863 (25.8)	1762 (26.3)	101 (19.3)	
Never	3,506 (53.4)	3,221 (52.9)	285 (59.3)	
Current	1,525 (20.8)	1,417 (20.8)	108 (21.4)	
Alcohol drink (%)				0.016
Former	1,334 (16.2)	1,233 (16.1)	101 (17.9)	
Heavy	1,434 (21.3)	1,350 (21.6)	84 (18.0)	
Mild	2,301 (36.348)	2,138 (36.4)	163 (36.2)	
Moderate	1,016 (16.3)	955 (16.6)	61 (13.0)	
Never	809 (9.8)	724 (9.4)	85 (14.8)	
Hypertension (%)	3,018 (37.6)	2,834 (38.0)	184 (32.7)	0.095
Diabetes Mellitus (%)	1,278 (13.7)	1,201 (13.9)	77 (11.4)	0.014
Daily sitting time				0.042
Short (%)	4,327 (58.9)	4,047 (59.3)	280 (53.7)	
Long (%)	2,567 (41.1)	2,353 (40.7)	214 (46.3)	
Sleep hours				0.012
Short (%)	2,688 (35.7)	2,470 (35.2)	218 (42.0)	
Long (%)	4,206 (64.3)	3,930 (64.8)	276 (58.0)	

BMI, body mass index; HbA1c, glycated hemoglobin; BUN, blood urea nitrogen; CVD, cardiovascular disease.

Laboratory findings showed that the constipation group had significantly lower albumin levels (42.371 ± 0.149 vs. 42.786 ± 0.074 g/L, *p* = 0.013), lower total bilirubin levels (0.718 ± 0.014 vs. 0.788 ± 0.006 mg/dL, *p* < 0.001), and lower uric acid levels (5.100 ± 0.078 vs. 5.560 ± 0.029 mg/dL, *p* < 0.001). Lifestyle characteristics revealed that the constipation group had a significantly higher proportion of never smokers (59.3% vs. 52.9%, *p* = 0.008) and never drinkers (14.8% vs. 9.4%, *p* = 0.016). Participants with constipation were more likely to have longer daily sitting time (46.3% vs. 40.7%, *p* = 0.042) and shorter sleep duration (42.0% vs. 35.2%, *p* = 0.012).

No significant differences were observed in WBC count (*p* = 0.434), BUN levels (*p* = 0.084), cardiovascular disease prevalence (*p* = 0.416), HbA1c levels (*p* = 0.845), race distribution (*p* = 0.183), or hypertension prevalence (*p* = 0.095).

### 3.2 Correlation between daily sitting time, sleep hours and constipation

**Figure 2:**
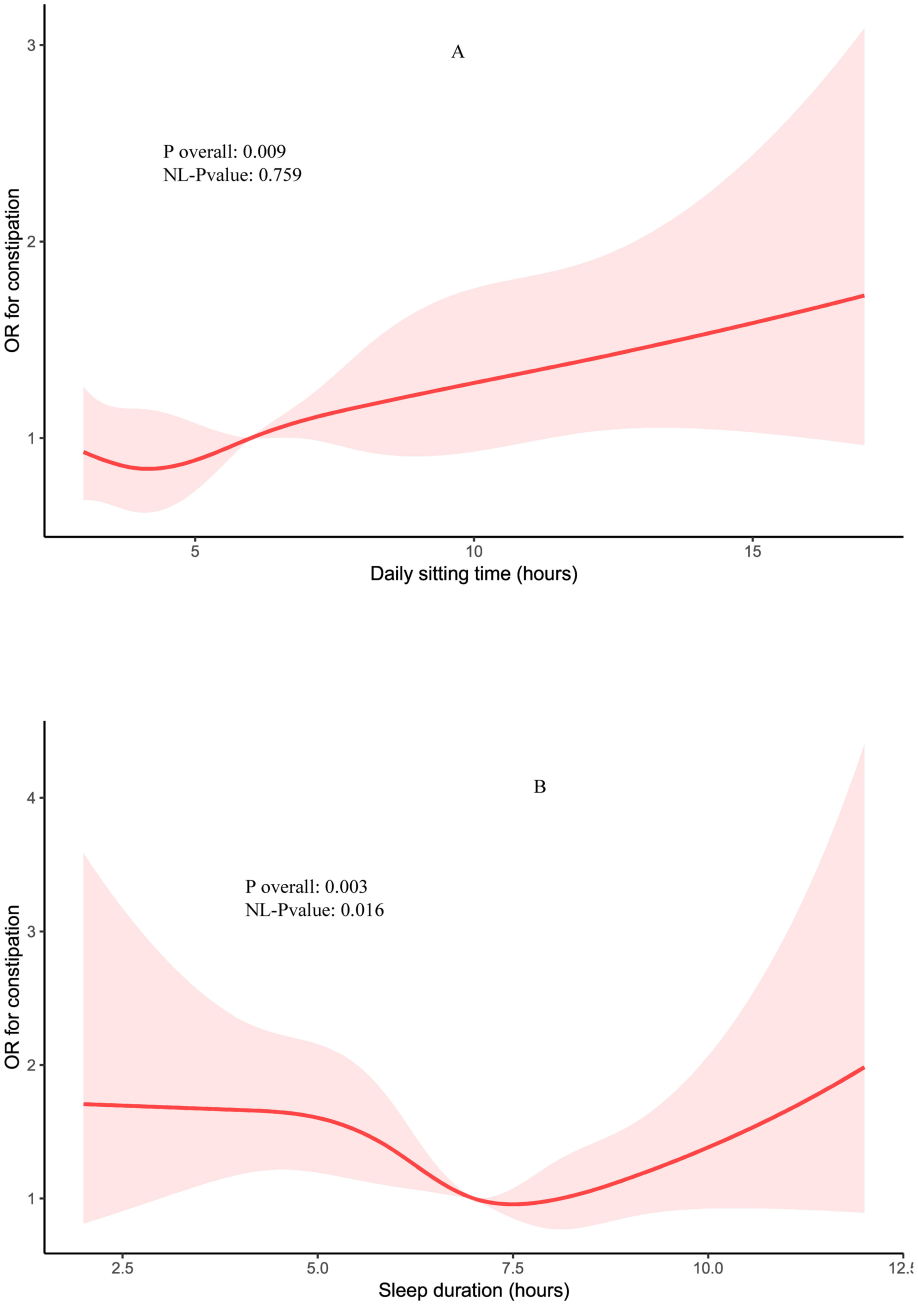
Restricted cubic spline (RCS) for the association between daily sitting time **(A)** and sleep hours with the risks of constipation **(B)**, after adjustment for age, sex, BMI, races, education, smoke, alcohol drink, diabetes mellitus, hypertension, poverty, uric acid, CVD, WBC and HbA1c.

The association between these factors and constipation was further evaluated using restricted cubic spline (RCS) curves, as shown in Figure 2. The RCS analysis revealed that daily sitting time, treated as a continuous variable, was associated with an increased adjusted risk of constipation, following a linear trend (Non-linear *p* = 0.759). In contrast, the relationship between sleep duration and constipation exhibited a significant non-linear pattern (Non-linear *p* = 0.016), suggesting that both short and long sleep durations may differentially impact constipation risk.

### 3.3 Associations of daily sitting time, sleep duration with constipation

A multivariable logistic regression analysis was performed to examine the associations of daily sitting time and sleep duration with constipation, as shown in Tables 2, 3. In the initial unadjusted model (crude model), participants with long sleep duration (≥7 h/day) had a significantly lower risk of constipation compared to those with short sleep duration (<7 h/day) (OR = 0.752, 95% CI: 0.604–0.936, *p* = 0.012). After adjusting for age, sex, and race in model 1, this inverse association remained significant (OR = 0.738, 95% CI: 0.581–0.936, *p* = 0.014). In the fully adjusted model (model 2), which accounted for BMI, education, smoking status, alcohol consumption, diabetes mellitus, hypertension, poverty, uric acid, cardiovascular disease, WBC, and HbA1c, participants with long sleep duration continued to exhibit a significantly lower risk of constipation (OR = 0.725, 95% CI: 0.553–0.952, *p* = 0.025).

**Table 2:** Association between the sleep hours and constipation.

	Crude model	Model 1^a^	Model 2^b^
Sleep hours			
Short	ref	ref	ref
Long	0.752 (0.604,0.936)*	0.738 (0.581,0.936)*	0.725 (0.553,0.952)*

^aModel 1 adjusted for age, sex, races.^

^bModel 2 adjusted for age, sex, BMI, races, education, smoke, alcohol drink, diabetes mellitus, hypertension, poverty, uric acid, CVD, WBC and HbA1c.^

*p < 0.05.

**Table 3:** Association between the daily sitting time and constipation.

	Crude model	Model 1^a^	Model 2^b^
Daily sitting time			
Short	ref	ref	ref
Long	1.257 (1.008,1.567)*	1.265 (1.013,1.580)*	1.424 (1.114,1.821)*

^aModel 1 adjusted for age, sex, races.^

^bModel 2 adjusted for age, sex, BMI, races, education, smoke, alcohol drink, diabetes mellitus, hypertension, poverty, uric acid, CVD, WBC and HbA1c.^

*p < 0.05.

Similarly, in the crude model, individuals with long daily sitting time (≥7 h/day) demonstrated a significantly higher risk of constipation compared to those with short daily sitting time (<7 h/day) (OR = 1.257, 95% CI: 1.008–1.567, *p* = 0.042). This association persisted in model 1 (OR = 1.265, 95% CI: 1.013–1.580, *p* = 0.038). In model 2, after full adjustment, the odds ratio further increased, indicating a stronger association (OR = 1.424, 95% CI: 1.114–1.821, *p* = 0.005).

### 3.4 Joint association and daily sitting time, sleep duration with constipation

We conducted a joint analysis to explore the combined effects of daily sitting time, sleep duration, and constipation. Interestingly, the results indicated that the combination of short sleep duration (<7 h/day) and long daily sitting time (≥7 h/day) was associated with the highest risk of constipation (Table 4). Specifically, compared to individuals with long sleep duration (≥7 h/day) and short daily sitting time (<7 h/day), those with short sleep duration and short sitting time had an odds ratio (OR) of 1.284 (0.996–1.656) for constipation risk. Additionally, individuals with long sleep duration and long daily sitting time had an OR of 1.336 (0.954–1.871) for constipation risk, indicating a notable interaction between these two factors.

**Table 4:** Adjusted OR and 95% CI for the associations of the daily sitting time and/or sleep hours with constipation.

	Crude model	Model 1^a^	Model 2^b^
Daily sitting time and sleep hours			
Q1	ref	ref	ref
Q2	1.238 (1.021,1.501)*	1.262 (1.036,1.539)*	1.284 (0.996,1.656)
Q3	1.178 (0.874,1.588)	1.186 (0.877,1.603)	1.336 (0.954,1.871)
Q4	1.696 (1.285,2.239)***	1.737 (1.294,2.332)***	1.975 (1.378,2.833)**

^aModel 1 adjusted for age, sex, races.^

^bModel 2 adjusted for age, sex, BMI, races, education, smoke, alcohol drink, diabetes mellitus, hypertension, poverty, uric acid, CVD, WBC and HbA1c.^

Q1: Sleep hours ≥ 7 h and daily sitting time < 7 h.

Q2: Sleep hours < 7 h and daily sitting time < 7 h.

Q3: Sleep hours ≥ 7 h and daily sitting time ≥ 7 h.

Q4: Sleep hours < 7 h and daily sitting time ≥ 7 h.

*p < 0.05, **p < 0.01, ***p < 0.001.

### 3.5 Subgroup analyses

The results of the subgroup analyses are presented in Table 5. Among participants with short sleep duration (<7 h/day) and long daily sitting time (≥7 h/day), the overall odds ratio (OR) for constipation was 1.932 (95% CI, 1.481–2.521). When stratified by age, participants over 60 years old had a significantly higher risk of constipation [OR, 2.716; 95% CI (1.139–6.477)], as did those under 60 years old [OR, 1.823; 95% CI (1.090–3.048)]. Additionally, participants with a BMI less than 30 [OR, 2.192; 95% CI (1.261–3.810)] and female participants [OR, 2.203; 95% CI (1.361–3.564)] were also found to have an increased risk of constipation under these conditions.

**Table 5:** Subgroups analyses of the effect of daily sitting time and sleep hours on constipation.

	Q1	Q2	Q3	Q4	P for interaction
Age					0.705
≥60	ref	1.272 (0.453, 3.574)	1.644 (0.669, 4.037)	2.716 (1.139, 6.477)	
<60	ref	1.293 (0.820, 2.039)	1.282 (0.722, 2.274)	1.823 (1.090, 3.048)	
BMI					0.457
≥30	ref	1.468 (0.630, 3.423)	1.463 (0.560, 3.822)	1.515 (0.622, 3.688)	
<30	ref	1.208 (0.745, 1.958)	1.265 (0.665, 2.406)	2.192 (1.261, 3.810)	
Sex					0.037
Female	ref	1.050 (0.722, 1.528)	1.596 (1.035, 2.460)	2.203 (1.361, 3.564)	
Male	ref	1.656 (0.790, 3.467)	0.893 (0.361, 2.211)	1.634 (0.621, 4.298)	

Q1: Sleep hours ≥ 7 h and daily sitting time < 7 h.

Q2: Sleep hours < 7 h and daily sitting time < 7 h.

Q3: Sleep hours ≥ 7 h and daily sitting time ≥ 7 h.

Q4: Sleep hours < 7 h and daily sitting time ≥ 7 h.

### 3.6 Interaction between sleep duration and daily sitting time on constipation

Results in Table 6 indicated that there was a non-significant synergistic effect of sleep duration and daily sitting time on constipation in Model 3 (adjusted RERI = 0.05 [−0.51, 0.61]; adjusted AP = 0.03 [−0.34, 0.28]; adjust SI = 1.06 [0.52, 2.16]).

**Table 6:** Association between sleep duration and constipation stratified by presence of short or long daily sitting time.

	Sleep duration≥7 h	Sleep duration<7 h	Effect of long sleep duration within the strata of long daily sitting time
	OR [95% CI]	OR [95% CI]	OR [95% CI]
Daily sitting time < 7 h	1 [Reference]	1.31 [1.02, 1.68]	1.31 [1.02, 1.68]
Long daily sitting time ≥ 7 h	1.48 [1.15, 1.9]	1.83 [1.39, 2.41]	1.24 [0.93, 1.66]
Multiplicative scale	0.95 [0.65, 1.39]		
RERI	0.05 [−0.51, 0.61]		
AP	0.03 [−0.34, 0.28]		
SI	1.06 [0.52, 2.16]		

## 4 Discussion

This study identified that both prolonged daily sitting time and insufficient sleep duration were linked to higher odds of constipation, especially among participants with a BMI of less than 30 and female participants. Additionally, the combination of excessive sitting time and short sleep duration was associated with the highest prevalence of constipation.

Our findings are consistent with a Hong Kong study, which reported that constipation was associated with excessive sedentary behavior (OR = 1.25) (25). The biological mechanisms linking prolonged daily sitting time and sleep duration to constipation are complex and multifaceted. Consistent with previous study, prolonged sitting is associated with reduced physical activity, which can lead to slower gastrointestinal motility (26). Decreased movement reduces the stimulation of the colon, leading to delayed transit time and increased water reabsorption from the stool, thereby resulting in harder, more difficult-to-pass stools characteristic of constipation. Additionally, sitting for extended periods can impair blood circulation, particularly in the abdominal area, which may further slow bowel movements (27). Studies have shown that physical inactivity is associated with metabolic disturbances, such as insulin resistance and increased inflammation, which could also contribute to gastrointestinal dysregulation (28). This sedentary behavior is not only harmful to metabolic health but can also disrupt the autonomic nervous system, further contributing to the development of constipation through decreased parasympathetic activity, which is crucial for normal bowel function.

In terms of sleep duration, both short and long sleep durations have been implicated in gastrointestinal disturbances, including constipation. A meta-analysis revealed that patients with a history of insufficient sleep were also associated with an increased risk of constipation (OR = 1.33) (2). Inadequate sleep can lead to alterations in autonomic nervous system regulation, increasing sympathetic activity and reducing parasympathetic tone, which are essential for promoting regular bowel movements. Short sleep duration may reduce skeletal muscle contraction, thereby increasing the risk of chronic constipation (29). In fact, chronic sleep deprivation or poor sleep quality has been shown to decrease muscle mass (30, 31) and lower rates of muscle protein synthesis (32). An unexplored hypothesis suggests that sleep disturbances might impair the function of the anal sphincter and increase pelvic floor muscle tension, exacerbating constipation symptoms. Chronic sleep deprivation has been associated with increased inflammation and oxidative stress, which could contribute to the impairment of smooth muscle function in the intestines (33). Additionally, circadian misalignment from irregular sleep patterns can disrupt gastrointestinal circadian rhythms, further exacerbating constipation risk (34, 35). These findings suggest that both sleep and sedentary behavior independently and synergistically affect gastrointestinal health, particularly in relation to bowel habits and constipation.

To better understand why the impact of prolonged sitting time and short sleep duration on constipation may be more pronounced in participants with a BMI <30 and among females, it is essential to explore the physiological and hormonal differences that contribute to this association. In previous research, the relationship between bowel symptoms and BMI has been a topic of debate. A case–control study of 96 irritable bowels symptom patients in Sweden found that higher BMI was associated with more severe symptoms, including bloating, gas, urgency, loose stools, and increased stool frequency (36). Conversely, a cohort study involving 958 Japanese adults suggested that lower BMI may serve as an indicator of elevated risk for constipation and hard stools (37). These conflicting findings underscore the complex and potentially bidirectional relationship between BMI and bowel health, suggesting that both extremes of BMI could be linked to different types of gastrointestinal issues. Moreover, women are more likely to undergo abdominal surgeries, which pose a higher risk of postoperative bowel dysfunction and can lead to chronic constipation (38). The presence of adhesions and weakened pelvic floor muscles after such surgeries can further contribute to the development of constipation. Additionally, lifestyle factors such as higher rates of sedentary behavior and psychosocial stressors, which are more prevalent among women, may amplify the observed sex-specific differences in constipation risk (39). Female sex hormones, particularly estrogen and progesterone, play a significant role in gastrointestinal motility and constipation risk (40). Estrogen has been associated with altered gut microbiota composition and bile acid metabolism, while progesterone slows colonic transit time, potentially contributing to constipation, especially in women (41). Moreover, fluctuations in these hormones during the menstrual cycle, pregnancy, and menopause may further modulate bowel function (42). These factors, along with the effects of prolonged sitting and inadequate sleep, may explain the increased prevalence of constipation in these subgroups.

There are limitations to consider. First, the findings are based on a United States cross sectional study and may not be directly generalizable to other populations. Then the observational design limits causal inference, and further interventional studies are needed to validate our findings. Third, it remains unclear whether prolonged sitting or short sleep duration directly causes constipation or if other unmeasured factors contribute to the association. Although we adjusted for multiple confounders, residual or unmeasured confounding (e.g., dietary fiber intake, stress levels) may still influence the results. Additionally, our definition of constipation relied on self-reported questionnaire data, which may be subjective. Future randomized controlled trials could offer more objective assessment methods, such as daily monitoring of bowel movements or the use of instruments to measure intestinal motility, enabling more precise follow-up.

## 5 Conclusion

In our sample, short sleep duration and prolonged daily sitting time were both associated with an increased risk of constipation. Additionally, the combination of short sleep duration and long daily sitting time exhibited the highest prevalence of constipation. These findings highlight the importance of adopting healthy sleep habits and reducing sedentary time to mitigate the risk of constipation. Future prospective cohort studies are necessary to further investigate and confirm this association.

## Data Availability

The original contributions presented in the study are included in the article/supplementary material, further inquiries can be directed to the corresponding author.
